# The National Association of Dental Faculties

**Published:** 1895-11

**Authors:** 


### Editorial.

### THE NATIONAL ASSOCIATION OF DENTAL FACULTIES.

Eleven years have passed into history since this body was organized in August, 1884. Its general work and the influence it has had in improving the standard of dental operations in this country is well known in all dental professional circles. It was called into being in April, 1884, by Professor R. B. Winder, of the Baltimore College of Dental Surgery, who, at that period, by correspondence with the Deans of the three dental schools in Philadelphia, gradually worked up an interest in the subject. It was no easy task to infuse his energy and enthusiasm into the minds of those first called in consultation. The effort to unify the dental profession seemed, at the time, a hopeless attempt. For years, in fact from the inception of the college idea and its practical illustration in opposing colleges, there had existed the same jealousy and mistrust of each other as formerly had an existence among the practitioners of the profession. While there was an honest effort to educate men, there was no standard by which the work could be regulated, or was there any disposition to unite to form one. The number of students in dentistry was limited prior to the enactment of laws governing it, and very few colleges were possessed with sufficient capital to secure the best ability in any direction. Up to the winter of 1876 it is safe to say that those who struggled with dental education did it at a great personal sacrifice of strength and pecuniary loss. In 1877 a change for the better was apparent, and classes grew rapidly, but no effort was made to improve the curriculum or to lengthen the course. Men were graduated in numbers who really had had but four months' instruction. They were taken for one session, beginning October 1 and ending, as far as instruction went, the first week in February, the balance of the time to the last of this month being devoted to examinations and preparation for commencement. Until the session of the "Faculties" held in August of the present year (1895), this has been the extent of the course of the largest number of the colleges of the country. It has now been increased to six months.

During the period from 1876 to 1884 a large proportion of graduates were sent out with their diplomas upon one session, superadded to a so-called five years' practice, and were entitled to class themselves as doctors of dental surgery, and to be respected as such. This would not have been quite so serious had the five years' practice been an honest labor for that time. With some it was, and with others more than five years could be placed to their credit. The mass of acceptances were, however, without doubt, based on statements impossible to verify. So much was this the case that the deans of the various schools made but little effort to look behind the certificates presented; indeed, such a course would have been impossible. While in many cases there was a laudable effort to stem this overwhelming tide of destructive influences, there was an element that seemed to make no effort to discover the truth whether or not the applicant for matriculation had had five years of practice or not. In illustration of this a case came to the writer from Europe. The woman claimed to have had five years of practice in Germany. This statement was at first credited, and she was taken as a private student by the writer. It was soon discovered that the five years' practice consisted in cleaning her infant children's teeth. She, of course, was dismissed, and, after futile attempts to enter a college in Philadelphia, she drifted, about January, to the West, and was graduated in March from one of the best of the Western schools with all the glory of a personal ovation.

These not infrequent occurrences became a scandal in the profession, but how to remove them, under the disturbing conditions mentioned, remained a problem.

These were some of the difficulties that confronted the conference in April, 1884. This was composed of Professor Winder, of the Baltimore College of Dental Surgery; Professor Garretson, of the Philadelphia Dental College; Professor Peirce, of the Pennsylvania College of Dental Surgery, and Professor Truman, of the

Department of Dentistry of the University of Pennsylvania. Each felt the seriousness of the situation, but the question was asked, "If we attempt this move will we be supported?" The answer was, "We will try;" and the call for a meeting of colleges was sent out to convene at the Sturtevant House, New York City, August 4, 1884.

It was with a feeling of much anxiety that this meeting was contemplated, and when the time came no one could prophesy with safety whether it would prove a success or a failure. It was in the nature of an experiment. Nothing that had ever been attempted before that came to a successful termination.

The surprise was great to find that the call had met with a response from eleven colleges, each of which had sent a delegate to represent it. Some of these were well known educators, others were strangers, but all were seemingly impressed with the importance of the work.

Probably no body ever met to organize in which the individual members were so much at a loss how to proceed as in this. No precedent existed for the formation of a constitution fitted for such a body to work under, and no one had any conception of the rules to be required for its government; in fact, it was unknown whether those present would even agree to form a temporary organization. This was, however, accomplished, and the basis of association was prepared and almost non-committal in its simplicity. It was felt, very justly, that, as this was an untried problem, we should let time and experience teach the members how to formulate rules necessary for their government. The wisdom of this has been more than justified. This meeting adjourned to meet at Saratoga Springs the same week, the American Dental Association having been called to meet at that place.

The most important action of this meeting, and that which stamped it as a body determined to begin earnestly, was the immediate effort to rid the profession at once and forever of the rule of five years' practice. This was done by a unanimous vote, and, although this was the first meeting of a society essentially full of crudities, so powerful was its immediate influence that all colleges, whether members or not, fell into line and adopted this measure of two full sessions.

It is unnecessary to follow this organization through the subsequent years to the present one of 1895. Its history is known, and its actions have been carefully watched and, at times, sharply criticised. It has shown the professional world that it could legislate and punish its members for violations of its rules. It has brought dental education to a high standard, and will carry it higher as the necessity occurs. It has led the way for the medical profession to organize a similar association,—indeed, before proceeding with their work, they were careful to secure from the writer full particulars of the organization in which we are at present interested. It advanced the curriculum of all the schools steadily and persistently. It forced all the dental colleges of the country to adopt a three years' course. It blocked the way for the conferring degrees *in absentia*, and insisted that no degree should be conferred except upon a basis of undergraduate work. It has done more than this, for it has made dental education worthy the respect of all intelligent men, so that to-day, after eleven years of work, dentistry stands as the peer of any of the special branches of the medical profession. The best men in the latter regard the time as not far distant when medical schools and medical departments of universities must meet the problem and answer the query, "How can dentistry be absorbed and become absolutely a part of medicine?" This cannot now be answered, but the time is not far away when it will press upon the attention of educators as well as upon professional thinkers.

Having thus run over the history of the work of the National Association of Dental Faculties, what now, may be asked, is its condition? We do not think it has reached its final period of usefulness. Indeed, it is believed it has more work yet to do than has been done in the past, valuable as that has been, but we approach the closing of the year 1895 with feelings not over hopeful.

The Association comprises within its fold two classes of workers, those connected with universities and those representing colleges organized from private funds and entirely sustained by the income derived from students. There is a middle class of colleges connected with, but not supported by, medical schools. These receive their support as the last mentioned.

The result of combining two seemingly antagonistic elements has, heretofore, not been harmful, as it has been to the mutual interest of all concerned to advance the standard and live up to it. There has been an earnest effort to respect the decisions of the main body.

The last meeting of this organization, held at Asbury Park last August, was the first in which two attempts were made, one successful, to lower the standard. The first was after voting a seven months' course to reconsider this action and change it to six, and the other was the conferring of an honorary degree, which the better sense of the meeting repudiated at a subsequent sitting.

The insidious element that seems to have entered this body is one that has wrecked others equally as useful,—the fell spirit of caucus, which was plainly manifested in the election of officers. This is the first time that this has been openly shown, and it marked a dividing line between the college and the university. If this be permitted there can be but one end, as was intimated in our article last month. The university representation *cannot consent to be sunk in the mire of an imperfect standard*, and we can assure the single colleges that they will not.

The *Western Dental Journal*, in a recent issue, somewhat severely took the Association to task for not advancing the standard by adding Latin and other higher branches to the preliminary examination, and also reflected on a certain member for his motion to lay the resolution on the table. From some knowledge of this person's motive in making this motion, it may be well to state, for the information of the editor, that the reason was not opposition to a move in this direction, for, on the contrary, he is fully alive to the necessity of a higher standard at the beginning of the work, but the time, in his opinion, has not yet arrived for this. It will be proper to concede it when the practical side of dental education has been fully satisfied by a full course of at least nine months, and a curriculum sufficient to meet all the demands of professional work.

In the opinion of some, we may have stated the case too frankly. Nearly a year still intervenes before another meeting. Let those interested look well to the future and to the interests of the dental profession that this cloud, now "not larger than a man's hand," be not increased until it overshadows and destroys the best organization ever planned for a beneficent purpose.

The remedy is easy. Let the separate colleges abandon at once the dangerous course seemingly adopted, and take immediate measures to advance the standard in all reasonable ways. With a moderate degree of wisdom infused into its deliberations this can be accomplished without friction, and then the Association of Faculties will live to lead the profession to higher things.

